# Supplementary material for: 3D micro-environment regulates NF-κβ dependent adhesion to induce monocyte differentiation
Source: Cell Death Dis. 2018 Sep 11;9(9):914. doi: 10.1038/s41419-018-0993-z (PMC6133927; doi:10.1038/s41419-018-0993-z)
Supplement: Supplementary file 1 — SUPPLEMENTAL MATERIAL [file 41419_2018_993_MOESM1_ESM.doc]

**Supplementary Material**

**3D micro-environment regulates NF−κβ dependent adhesion to induce monocyte differentiation**

Running Title: Adhesion induces differentiation

Anindita Bhattacharya^†^, Mahesh Agarwal^†^, Rachita Mukherjee, Prosenjit Sen, Deepak Kumar Sinha^*^

Department of Biological Chemistry

Indian Association for the Cultivation of Science

Jadavpur, Kolkata -700032, India

^†^ These authors contributed equally to this work

^*^Corresponding author, Email: [bcdks@iacs.res.in](mailto:bcdks@iacs.res.in) / [emaildks@gmail.com](mailto:emaildks@gmail.com)

##### Phone: +91 334734971 (Ext 1106),

##### Fax: +91-33-2473 2805


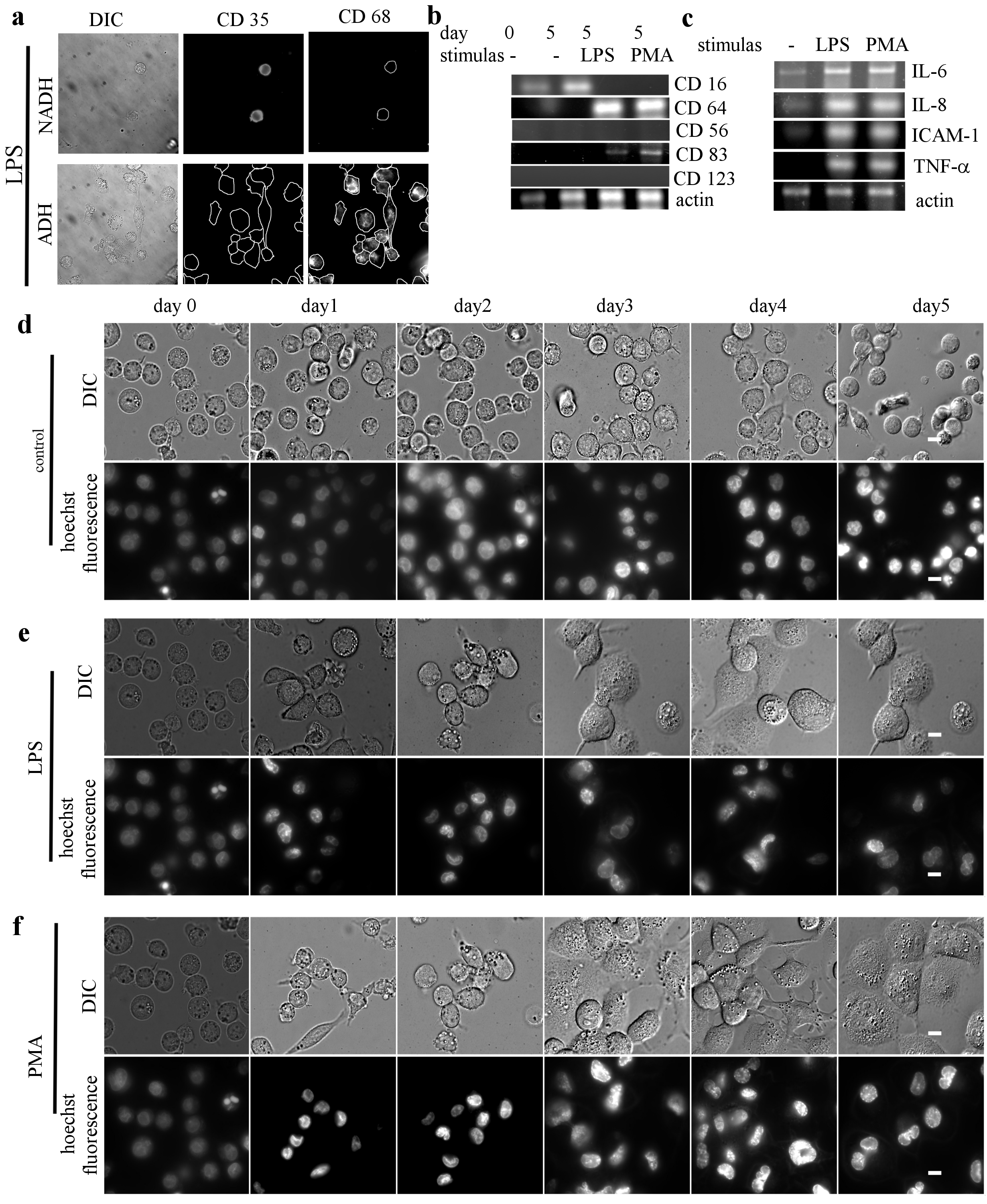


**Fig. S1: Chemical stimulation of monocyte cells gives rise to two populations of cells with distinct phenotypes** .**a**, DIC (left), immunostain of CD35 (middle) and immunostain of CD68 (right) images of the LPS treated non-adherent (NADH-THP-1) (upper panel) and adherent (ADH-THP-1) (lower panel) fraction of THP-1 cells. The images are taken on 5^th^ day post induction/seeding. **b**, semi-quantitative RT-PCR of CD16, CD64, CD 83, CD123 and CD 56 on THP-1 cells cultured in different induction conditions. **c,** Semi-quantitative RT-PCR analysis of IL-6, IL-8, ICAM-1, TNF-α of THP-1 cells cultured in different induction conditions. **d-f**, Cellular (DIC) and the nuclear (fluorescence, Hoechst stained ) morphology of control (b), LPS (c) and PMA (d) treated cells from day 0 to day 5.


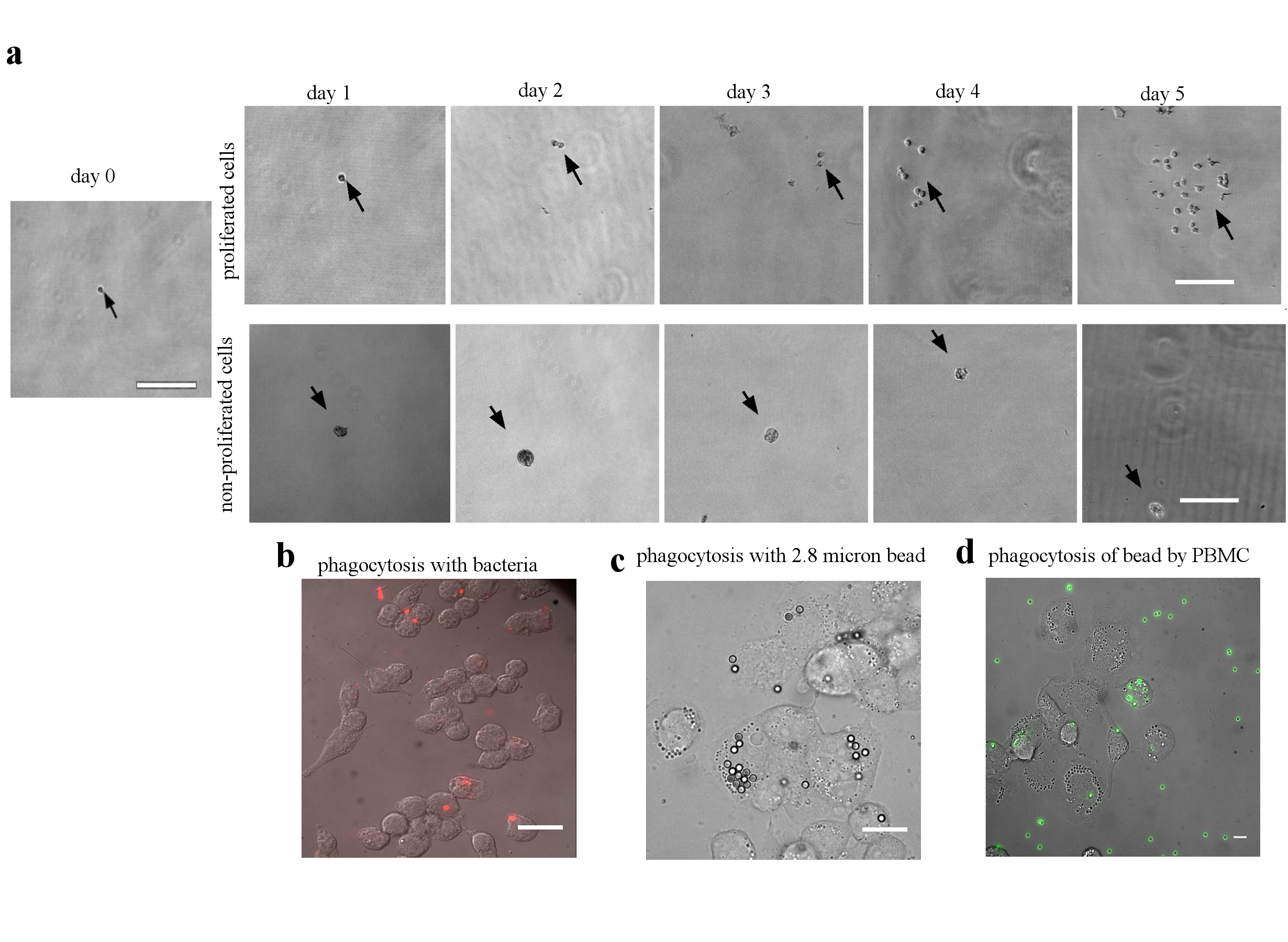


**Fig. S2: An adherent subpopulation of cells functionally resemble macrophages**. **a**, Phase contrast images of PMA treated single THP-1 cell (SCPA) from day 0 to day 5. Upper panel depicts the proliferating cells while the lower panel depicts the non-proliferating cells in response to inducer treatment. **b**, Super imposed images of THP-1 cells (DIC) and phagocytized RFP tagged *E.coli.* **c**, DIC images of THP-1 cells engulfing 2.8μm bead. **d,** Super imposed images of PBMCs (DIC) and phagocytized green fluorescent beads.


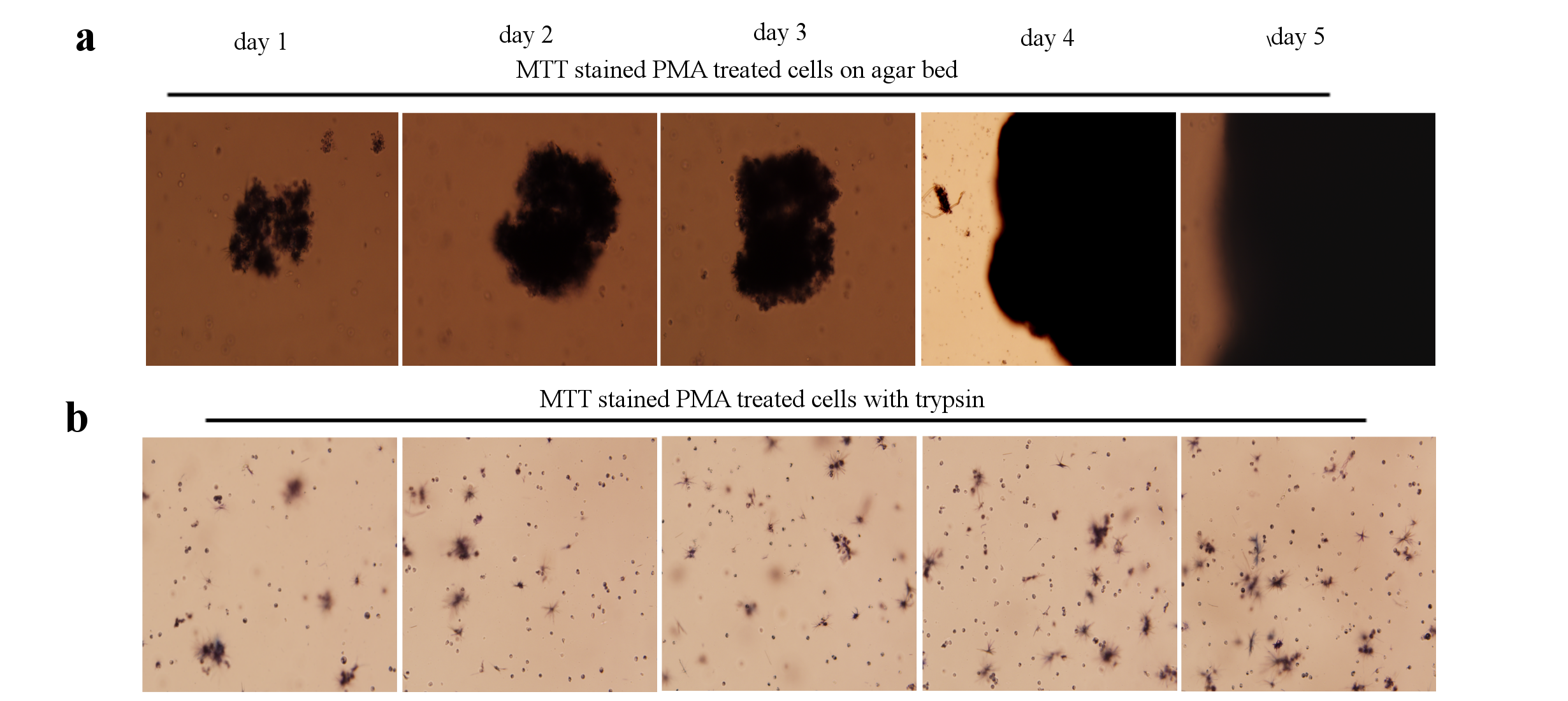


**Fig. S3: Adhesion is both necessary and sufficient for monocyte to macrophage differentiation in 2D culture.** **a-b**, Bright field images of THP-1 cells treated with PMA on day 0 and stained with MTT on day 1 to day 5 in adhesion incompatible conditions such as on agarose bed (a) or on collagen treated glass surface in presence of 0.25% trypsin (b).


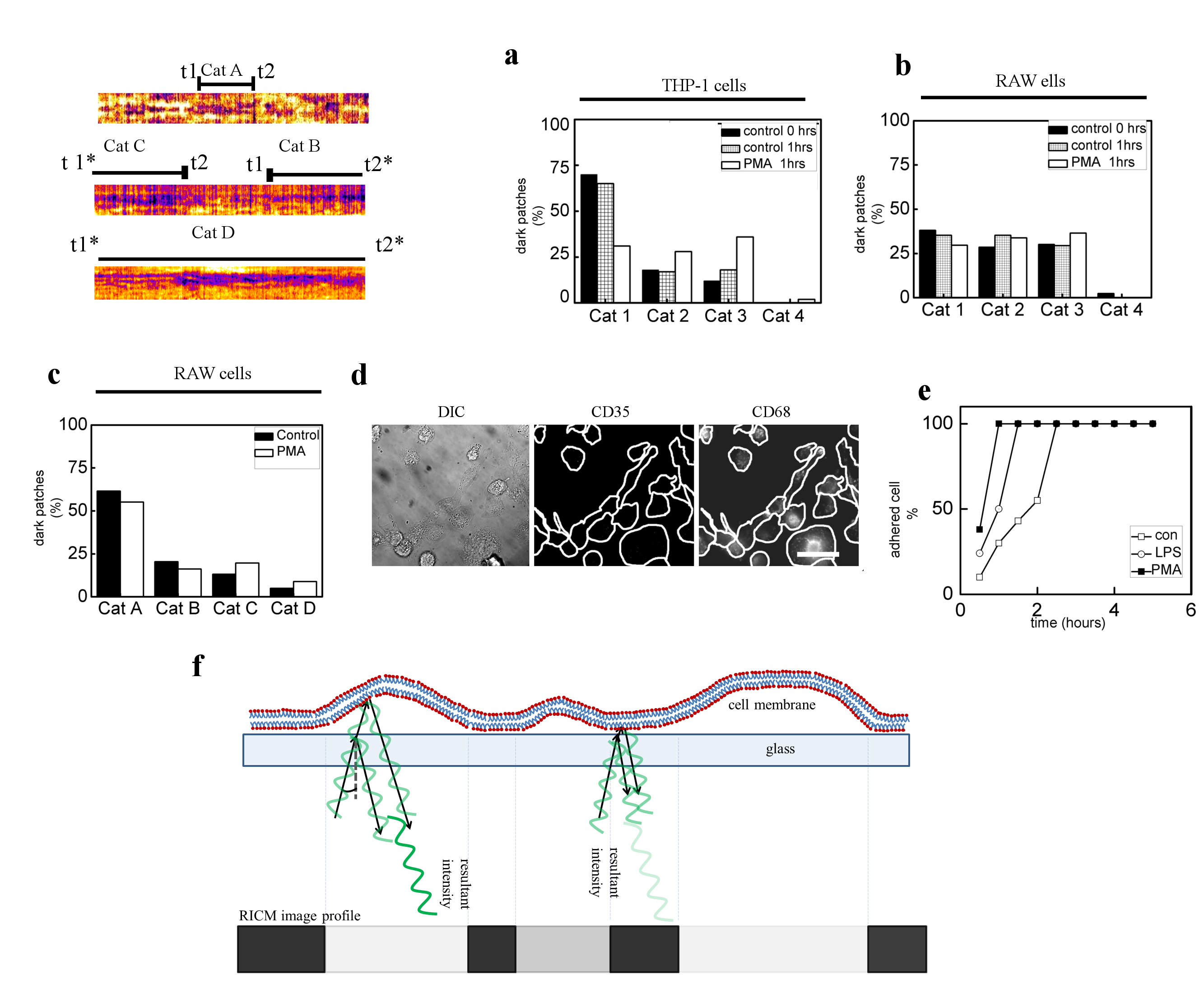


**Fig. S4: Chemical inducer alters membrane fluctuation to facilitate adhesion.** Notation depicting parameter for classification for different categories of dark puncta in figure a-c. **a**, Plot depicting the quantification of different categories of dark puncta in THP-1 cells under different conditions. **b**, Plot depicting the quantification of different categories of dark puncta in RAW cells under different conditions. **c**, Plot depicting the quantification of different categories of focal adhesion in RAW cells under different conditions.**d**, DIC (left), immunostain of CD35(middle) and immunostain of CD 68(right) images of RAW cells. **e**. Quantification of percentage of adhered RAW cell at different time post seeding with and without chemical stimulus. **f,** Cartoon diagram of RIM

| Catagory | Time of Appearance | Time of Disappearance |
| --- | --- | --- |
| A | Known | Known |
| B | Known | Unknown |
| C | Unknown | Known |
| D | Unknown | Unknown |

Supplementary note for figure S4:-We have categories the dark patches based on their appearance and disappearance time.The patches whose forms and disappear and whose exact duration of occurance is known falls into category A. The newly formed patches those does not disappear with time falls into catregory B. The already existing patches those dissapear with time falls into categoty C. @Patches that exist and remain stable throughout the entire duration falls into category D. we have summeries the catagories in the following table.


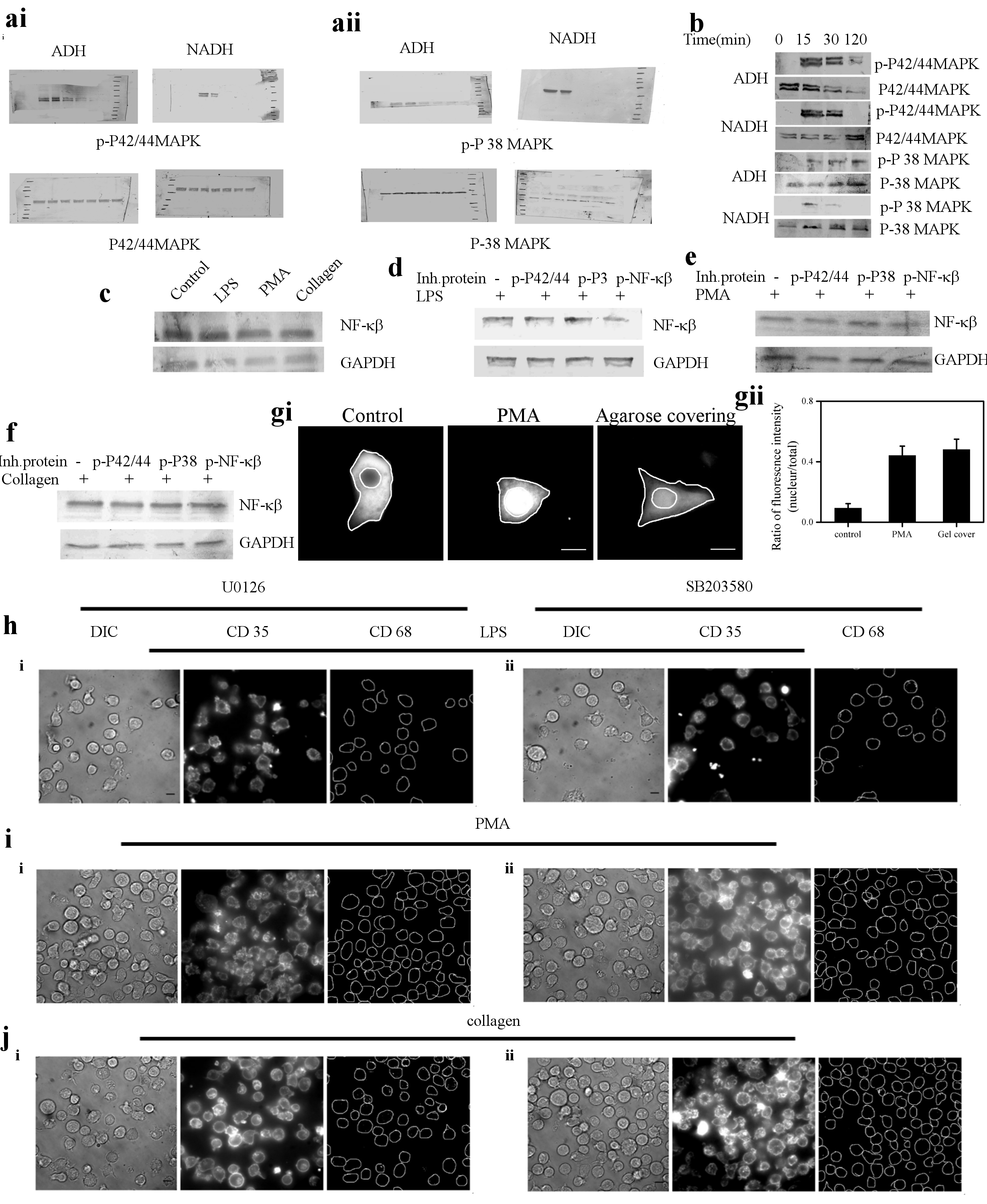


**Fig. S5: Adhesion dependent activation of NF-κβ is responsible for differentiation** **a** , Raw Western blot images phospho (upper panel) and non phospho (lower panel) P38 MAPK (ai) and P42/44 MAPK (aii) of THP-1 cell seeded in adherent and non adherent conditions after 0,15,30,120,240,480 and 720 minutes (lane 1-7) post PMA induction. **b,** Western blot images phospho P38 MAPK and phospho P42/44 MAPK of PBMCs seeded in adherent and non adherent conditions after 0,15,30,120minutes post PMA induction. **c,** Western blot of NF-κβ in the whole cell lysate of populations of THP-1 cells in different conditions. **d-f**, Western blot of NF-κβ in whole cell lysate of THP-1 cells stimulated with LPS (d), PMA (e) and collagen-coated surface (f) under different conditions of inhibitor treatment (U0126: P42/44MAPK, SB203580: P38 MAPK and NF-κβI: NF-κβ) (Inh: Inhibited). **g,** Fluorescent images of NF-κβ EGFP transfected RAW cells at different conditions (gi) and quantification of fluorescent intensity in nucleur to total cells (gii). Cell and nucleur boundary are indicated by white lines. **h-j,** DIC (left column), immunostain images of CD35 (left column) and CD 68 (Right column) of THP-1 cells treated with U0126 (hi, ii, ji) and SB203580 (hii, iii, jii) under stimulation with LPS (h) PMA (i) collagen-coated surface (j). Scale bar 10 μm.

**
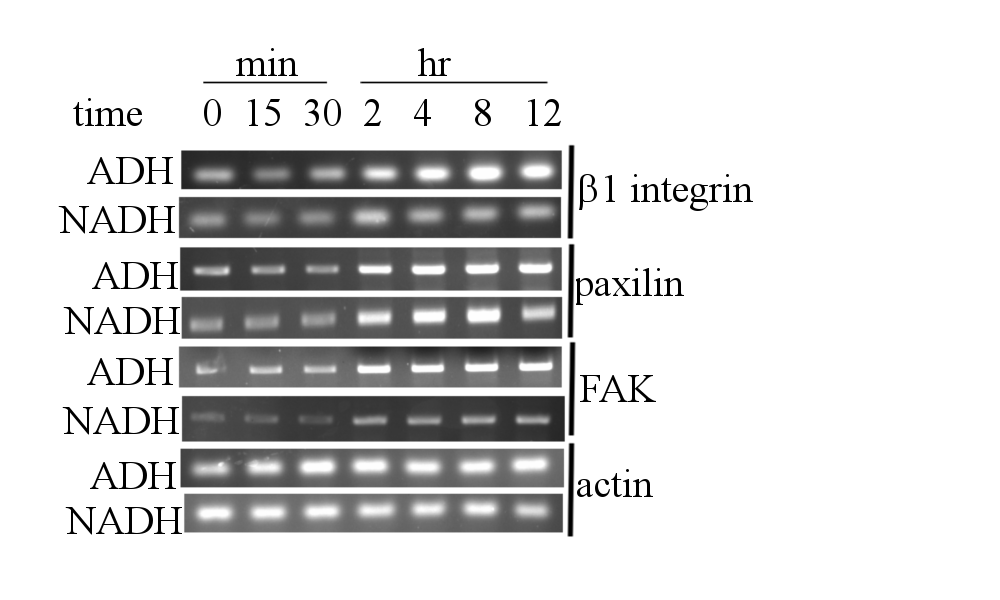
**

**Fig. S6: Adhesion mediated positive feedback loop regulates the p-NF-κβ levels.** Semi-quantitative RT-PCR of adhesion genes (β1 integrin, Paxilin and FAK) in THP-1 cells at different time post induction with PMA and seeded on glass surface and agarose bed.

**
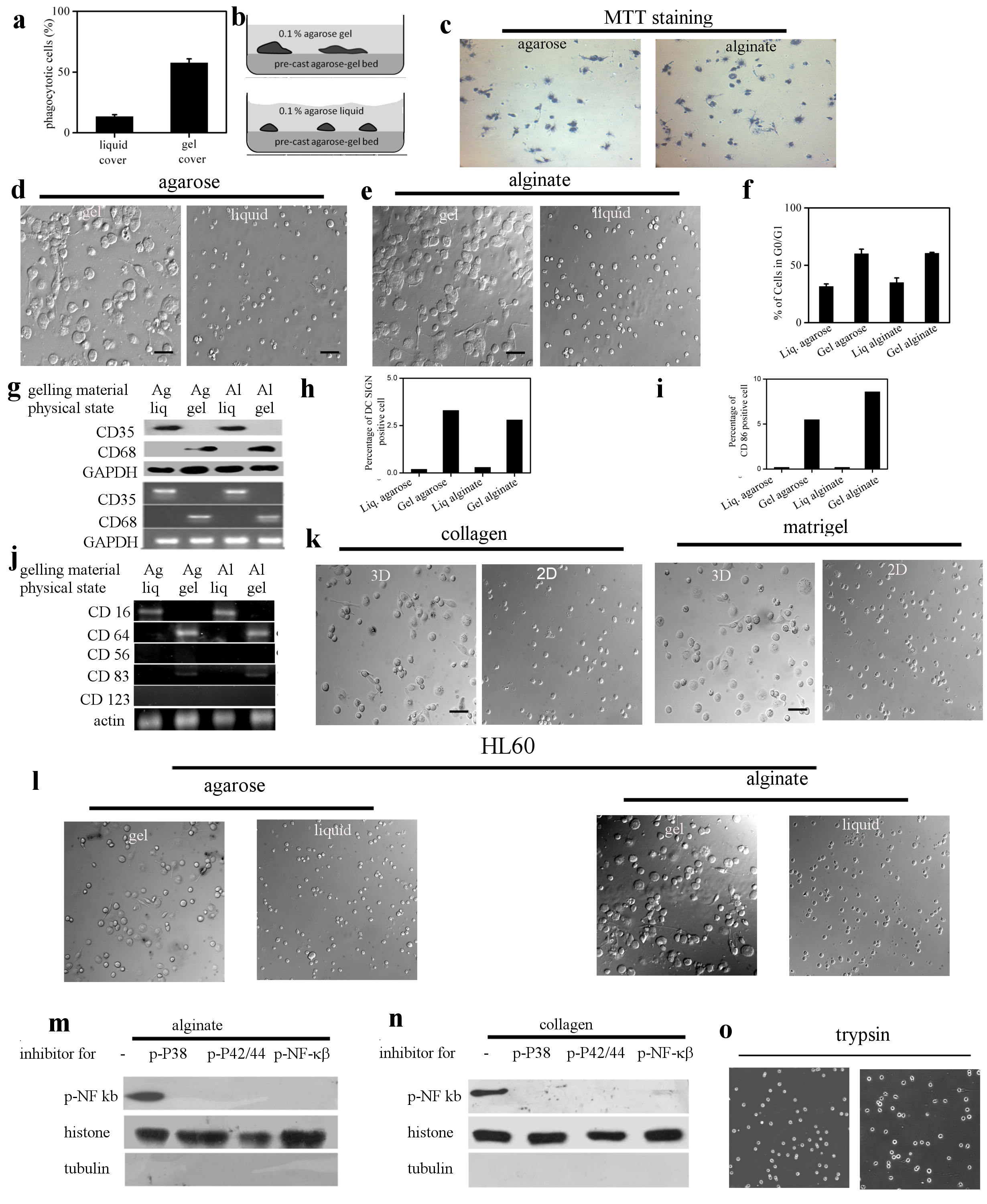
**

**Fig. S7: 3D gel-like microenvironment induces adhesion to facilitate differentiation.** **a**, The percentage of phagocytotic THP-1 cells cultured with cover of identical chemical composition (0.1% agarose) in liquid and gel state. **b**, The schematics of the method of covering THP-1 cells with agarose gel/liquid on substrate precoated with agarose gel. **c**, Bright field images of MTT stained THP-1 cells on day 5 cultured in the gel like microenvironment of agarose (left panel) and alginate (right panel). **d**, DIC images of THP-1 cells in RPMI media on day-5 cultured in 0.1% agarose liquid (left panel) or gel (right panel) like micro-environment. **e**, DIC images of THP-1 cells in RPMI media on day-5 cultured in 1% alginate liquid (left panel) or gel (right panel) like micro-environment. **f**, Plot depicting the percentage of G0/G1 arrested cells isolated from liquid or gel like micro-environment of agarose or alginate. **g**, Western blot (upper) and semi-quantitative RT-PCR (lower) analysis of CD35 and CD68 on THP-1 cells cultured in different gelling medium (Ag: agarose, Al: alginate) under different physical state (gel: 3D gel; liq: liquid). **h-i**, FACS analysis of DC SIGN and CD 86 stained cells isolated from liquid or gel like micro-environment of agarose or alginate. **j**, Semi-quantitative RT-PCR analysis of CD16, CD64, CD 83, CD123 and CD 56 on THP-1 cells cultured in different gelling medium (Ag: agarose, Al: alginate) under different physical state (gel: 3D gel; liq: liquid). **k**, DIC images of THP-1 cells cultured in 2D (left panel) or 3D (right panel) collagen and matrigel matrix. **l**, DIC images of HL-60 cells in RPMI media on day-5 cultured in liquid (left panel) or gel (right panel) like micro-environment of 0.1% agarose and 1% alginate (bottom panel). **m-n**, Western blot of p-NF- κβ in the nuclear fraction of THP-1 cells isolated from alginate (k) and collagen (l) gel treated with SB203580 (p-P38 MAPK), U0126 (p-P42/44 MAPK) and NF- κβ (NF- κβI nuclear translocation) inhibitors. **o**, Phase contrast images of THP-1cells treated with trypsin in serum free conditions and cultured in agarose (left panel) alginate(right panel) gel.


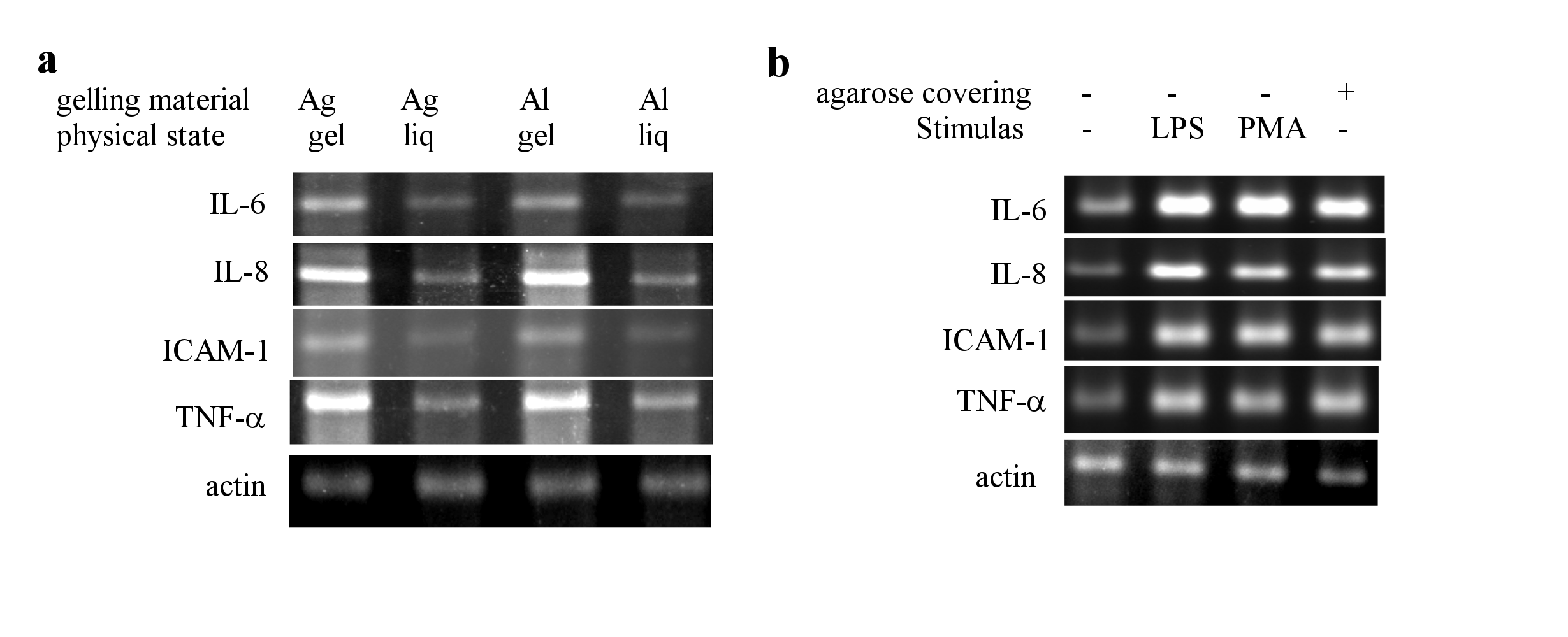


**Fig. S8: 3D gel-like microenvironment induces NF-kb target genes upregulation.** **a**, Semi-quantitative RT-PCR analysis of IL-6, IL-8, ICAM-1, TNF-α of THP-1 cells cultured in different gelling medium (Ag: agarose, Al: alginate) under different physical state (gel: 3D gel; liq: liquid).**b**, Semi-quantitative RT-PCR analysis of IL-6, IL-8, ICAM-1, TNF-α of PBMC cultured in different conditions.

**Appendix-1**

Chemical stimulus like PMA/LPS induces an adhesion simultaneously in every THP-1 cells. Therefore the process of differentiation is synchronized within the narrower time window. However, in the case of collagen-induced nonspecific adhesion, the differentiation of THP-1 cells is comparatively desynchronized leading to lesser fraction of phagocytotic cells.

**Appendix-2**

Reflection Interference Microscopy (RIM): RIM is used to measure small inter-surface separation. In biology it helps to study membrane dynamics close to surface, especially in context of cell adhesion. RIM provides sharp and high contrast images of focal adhesion of cells grown on the glass surface. This techniques work on reflection of incident beam light as it passes through material of different refractive indices. In case of cells on glass surface, the reflected beams produces either constructive or destructive interference depending upon the distance of cell membrane from the glass surface. Membrane near to the glass surface results in destructive interference whereas membrane at a distance causes constructive inference, which results in dark and bright patches in a images. So based on the intensity of RIM image one can infer the distance between the glass surface and cell membrane.

Supplement Table 1

| Primers | Sequence |
| --- | --- |
| CD 35 F | GGAAAGCCTTTGGAATAGCA |
| CD35 R | GGTTTTCCTGTGTGTCTCCC |
| CD 68 F | TTCACCAGCTGTCCACCTC |
| CD 68 R | CACTGGGGCAGGAGAAACT |
| CD 16 F | GCTCCTCCCAACTGCTCTGCTAC |
| CD 16 R | ATGAAGTAGCTCGAGGCCTGGC |
| CD 64 F | GCACAGCCACTCAGACCTCGAC |
| CD 64 R | CACATTGTACACCAGCTTATCCTTCC |
| CD 83 F | TGGAGGGTGGTGAAGAGAGGATG |
| CD 83 R | AGTCTCTTCTTTACGCTGTGCAGGG |
| CD 123 F | CTCAAAAGTTCCCACATCCTGGTGC |
| CD 123 R | CTCTGACCTGTTCTGTGATTACAGGCTG |
| CD 56 F | GAAAACAGATGAGGGCACTTATCGCTG |
| CD 56 R | TGTTCCCCATCCTTTGTCCAGCTC |
| ICAM F | ACTGGCACCCCTCCCCTCT |
| ICAM R | TGGCTCCATGGTGATCTCTCC |
| E-cad F | CGGGAATGCAGTTGAGGATC |
| E-cad R | AGGATGGTGTAAGCGATGGC |
| Actin F | GAAGCATTTGCGGTGGACCAT |
| Actin R | TCCTGTGGCATCCACCAAACT |
| β1 integrin F | CAGCTCTTGGAATAGACACAGCAAG |
| β1 integrin R | GCTTAAATTTCCTCGGTTATAGCTGC |
| Paxillin F | GCAGGTTGTGACCGCCATGGG |
| Paxillin R | GTCCTTCTCGTGGAACCCTTCGG |
| FAK F | AGATGTCCAGTAAAATCCAGCCAGCC |
| FAK R | GTCTTGCTTGGTCAATGACATCGAGTAAG |
| i-NOS F | CGAGATCAATGCAGCTGTGCTCC |
| i-NOS-R | CGTAGTTCAACATCTCCTGGTGGAAC |
| TNF-α F | CGCTCTTCTGCCTGCTGC |
| TNF-α R | CCACCAGCTGGTTATCTCTCAG |
| IL-8 F | TTTTGCCAAGGAGTGCTAAAG |
| IL-8 R | AACCCTCTGCACCCAGTTTTC |
| IL-6 F | ACTCACCTCTTCAGAACGAATTG |
| IL-6 R | CCATCTTTGGAAAGGTTCAGGTG |

Supplement Table 2

| Antibody | Company |
| --- | --- |
| CD 35 | abcam |
| CD 68 | abcam |
| GAPDH | Sigma |
| p-P38 MAPK | Cell signaling technologies |
| P38 MAPK | Cell signaling technologies |
| p-P42/44 MAPK | Cell signaling technologies |
| P42/44 MAPK | Cell signaling technologies |
| p-NF-κβ | Cell signaling technologies |
| NF-κβ | Cell signaling technologies |
| Histone | sigma |
| tubulin | Cell signalling technologies |
| DC SIGN | BD bioscience |
| CD 86 | BD bioscience |
